# *SHADOW3*: a new version of the synchrotron X-ray optics modelling package

**DOI:** 10.1107/S0909049511026306

**Published:** 2011-07-20

**Authors:** Manuel Sanchez del Rio, Niccolo Canestrari, Fan Jiang, Franco Cerrina

**Affiliations:** a European Synchrotron Radiation Facility, 6 Jules Horowitz, 38000 Grenoble, France; b Institut Louis Néel, CNRS, Grenoble, France; c Electrical and Computer Engineering, Boston University, 8 St Mary’s Street, Boston, MA 02215, USA

**Keywords:** *SHADOW*, ray-tracing, X-ray optics

## Abstract

A new version of the popular X-ray tracing code *SHADOW* is presented. An important step has been made in restructuring the code following new computer engineering standards, ending with a modular Fortran 2003 structure and an application programming interface (API). The new code has been designed to be compatible with the original file-oriented *SHADOW* philosophy, but simplifying the compilation, installation and use. In addition, users can now become programmers using the newly designed *SHADOW3* API for creating scripts, macros and programs; being able to deal with optical system optimization, image simulation, and also low transmission calculations requiring a large number of rays (>10^6^). Plans for future development and questions on how to accomplish them are also discussed.

## Abstract 2

*SHADOW3*, a new version of the X-ray tracing code *SHADOW*, is introduced.

## 1. Introduction

One step before the construction of any X-ray instrument, such as a synchrotron beamline, is the accurate conceptual design of the optics. The beam should be propagated to a given image plane (usually the sample position) and its characteristics should be adapted to the experimental requirements in terms of flux, monochromatization, focal dimensions, *etc*. The designer’s goal is not only to verify compliance to a minimum set of requirements, but also to find an optimum matching between the source and the optics phase space to obtain the best possible performances.

*SHADOW* is a widely used program for the simulation of optical systems, specifically geared to the synchrotron radiation domain. It is based on a geometrical ray-tracing approach, but also traces field amplitude with phase difference, and therefore wave features beyond the validity domain of geometric optics. This is called the phase ray-tracing method (Lee & Zhang, 2007 ▶). While there are many optical ray-tracing programs, *SHADOW* is unique because of its special focus on synchrotron radiation. This is evident in the interface, but is also present in its core or engine where models are built to specifically address problems of synchrotron radiation beamlines such as crystal diffraction, glancing optics and photon energies in the X-ray range. In fact, the code *SHADOW* has become the *de facto* standard for synchrotron radiation ray-tracing calculations because it is flexible and capable of adapting to any beamline configuration. It has also demonstrated its reliability during more than 25 years of use, as shown in hundreds of publications. Ergo, it is relatively simple to use and well documented, and is freely available (open source).

Indeed, almost all of the synchrotron beamlines today in existence have in some way benefited from the help of *SHADOW*. In most facilities a large number of beamline optics have been designed and verified using the program, including applications in: mirror optics, from microscopes to X-ray lithography beamlines; grating monochromators, fixed- and variable-line spacing; capillary optics, crystal optics, both in reflection and transmission, and polarized sources and polarization transfer.

## 2. History of *SHADOW* and the birth of *SHADOW3*

The birth of *SHADOW* is linked to the first dedicated use of synchrotron radiation at the University of Wisconsin during 1965–1967. A team led by particle physicist Ednor Rowe built Tantalus. He quickly adapted the machine to make synchrotron radiation and soon the facility was crowded with experimentalists from all over the world. In 1977, Aladdin, a new and larger synchrotron radiation source, began construction. *SHADOW* was born during the Tantalus–Aladdin transition with a clear scientific motivation: Monte Carlo simulation of X-ray optical systems, in particular grating monochromator design (toroidal grating monochromator and the extended range grasshopper) and reflection by toroidal and spherical mirrors. The requirements were ambitious enough to promote *SHADOW* to a universal level independently of Aladdin’s beamlines. Among these requirements one can mention: accuracy and reliability, ease of use, flexibility, economy of computer resources, VAX-11 computers, efficient Monte Carlo approach, use of reduced number of rays, exact simulation of synchrotron radiation sources, use of vector calculus for directions and operations rather than angles and trigonometry, implementation of a structured code, easy-to-use user-interface, and to be available to users. It took about two years of development to finish the first *SHADOW1* version, written in Fortran 77 with deep usage of the VAX/VMS extensions. The program was introduced in 1982, and has continued growing since. A first publication (Cerrina, 1984 ▶) explained the main philosophy of the new ray-tracing code and its application to grating monochromators. Between 1984 and 1990, *SHADOW* was exclusively used in the VAX-VMS mainframes, and the code was distributed in magnetic tapes sent by post. C. Welnak supported the increasing users community, by writing documentation and debugging, *etc. SHADOW* was upgraded to include new models with the help of F. Cerrina’s students: B. Lai (Lai *et al.*, 1988 ▶, 1989 ▶), K. Chapman (Chapman *et al.*, 1989 ▶), G. J. Chen (Chen, Cerrina *et al.*, 1994 ▶; Chen, Guo *et al.*, 1994 ▶), S. Singh (Singh *et al.*, 1996 ▶), *etc*. Some papers on *SHADOW* and its upgrades appeared (Lai *et al.*, 1988 ▶, 1989 ▶; Welnak *et al.*, 1992 ▶, 1994 ▶). Other scientists collaborated with F. Cerrina to develop models for new optical elements, like J. Underwood for multilayers or M. Sanchez del Rio for several types of crystals (Sanchez del Rio *et al.*, 1992 ▶, 1994 ▶; Sanchez del Rio & Cerrina, 1992 ▶).

During the early 1990s the use of VAX-VMS mainframes started to decline, with the incipient use of UNIX workstations for scientific computing. *SHADOW* required a significant remodelling and restructuring to run with the new machines. M. Khan performed this conversion and *SHADOW2* was born. This version was completed by a new user interface and new graphic tools based on the *PLPLOT* libraries, replacing the TopDrawer library in VAXes. During this period *SHADOW* was essential for the development of the third-generation X-ray sources, in particular at the ESRF. A new complete and independent graphical user interface (GUI) called *ShadowVUI* was developed at the ESRF, and it was soon made available to the user community through the X-ray optics toolbox *XOP* (Sanchez del Rio & Dejus, 2004 ▶). During this time some tools for mirror roughness (Singh *et al.*, 1996 ▶) or a pre-processor for slope errors (Sanchez del Rio & Marcelli, 1992 ▶) were also developed.

At the turn of the millenium, *SHADOW* continued to give services using the *SHADOW2* kernel. Much of the graphical tools and post-processors were replaced by the ones built with the *ShadowVUI* interface. The fact that it is still widely used today is a testament to the original architecture of the program, created with extensibility and accuracy as the main goals. As the field of synchrotron radiation instrumentation advances, the requirement on the optics become more stringent. Today, third- and fourth-generation sources often use diffraction-limited optics in a broad domain of wavelengths, from the infrared to hard X-rays. There is a clear need for an optical modelling tool capable of simulating not only the behaviour of an ideal optical system but also the effect of imperfections, misalignments and other issues found in real beamlines.

Today’s computers are very powerful tools, and easily surpass the old mainframes. Thus, it is important to take advantage of new possibilities for performing studies and analysis that would have been impossible on machines with lower speed and smaller memory. It was becoming more and more urgent to update *SHADOW* because: the binaries available presented problems in new machines using new libraries; the current version became heavy and hard to recompile (*e.g.* the g77 compiler used to build *SHADOW2* is no longer supported); some limitations that were inherited from the old computers (like the limitation in number of rays) became important drawbacks; and it became difficult to add new features owing to an old structure that became complex after the many upgrades over more than 20 years.

The path towards *SHADOW3* was discussed on several occasions, but no commitment was made because of the lack of manpower and funds. It is notable that *SHADOW*, together with its *ShadowVUI* interface available today, was created and developed without its own budget. It used resources for the beamline design and construction of Aladdin and other synchrotrons including the time and dedication of some of the authors through their institutions (like the ESRF). The birth of *SHADOW3* was fuelled by the ESRF Upgrade Programme 2008–2017. The design and construction of the new beamlines require a powerful ray-tracing tool, and *SHADOW* still was the most advanced X-ray tracing code for that. M. Sanchez del Rio discussed this issue in 2008 with F. Cerrina, defining an upgrade planning with clear requirements, specifications and time scheduling, that ends now with *SHADOW 3.0* .

## 3. Specifications and implementation

From the user point of view, the *SHADOW* package can be divided into several parts:

(i) A kernel consisting of two main programs, one for calculating the sources (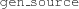) and a second one for tracing this source though the beamline (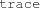).

(ii) A set of pre-processors linked to an optical library (X-ray cross sections) for dealing with reflectivity and transmission of mirrors (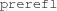), multilayers (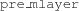) and crystals (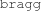). Another useful pre-processor is used for preparing mesh surfaces (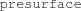).

(iii) A set of utilities to visualize and analyze results files (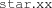, 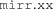, 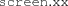 files), such as 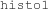, 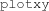, 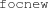, *etc.*

(iv) Other utilities (graphic libraries, menu, *etc.*).

(v) Graphical user interface.

The key part of *SHADOW* is its kernel, consisting of two main programs: 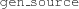 and 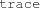. A minimum set of utilities were also upgraded, and others that could be replaced by external user interfaces were suppressed. The GUI, the graphics based on *PLPLOT*, and the terminal *MENU* are no longer supported (these are very complex and system-dependent). The *SHADOW* primer, a useful manual for getting started with *SHADOW*, has been updated for *SHADOW3*.

The update of *SHADOW* was evaluated facing the possibility of rewriting everything from scratch, perhaps using a new language. However, this option was discarded because of the inability to meet the resources needed; for example, the lack of specialized man-power to write, review and test the new version.

It was decided to rebuild *SHADOW* using the new Fortran standards, to match important requirements:

(i) Back-compatibility, meaning that *SHADOW*’s users will feel ‘comfortable’ with the new version, and files created by the old version are accepted by the new one.

(ii) Solve important limitations of the old versions, such as the limitation in number of rays.

(iii) Flexibility: adding and modifying features must be easier, helped by a simple compilation mechanism.

(iv) Interoperability: *SHADOW* should be callable from an API. The advanced user or programmer can easily modify the main code and create *ad hoc* codes. The API also will allow the possible use of different GUIs, implementation of new ones and also the integration of *SHADOW* into other packages.

The structured code of *SHADOW2*, split into many Fortran functions and subroutines, allows the re-use and reorganization of most routines and code parts. In addition, the original architecture of *SHADOW* is well suited to the new modular programming typical of modern languages. The Fortran 77 common blocks used massively in *SHADOW2* have been completely removed, and replaced by global variables within Fortran 90 modules, in accordance with modern programming recommendations. Changes and improvements are still needed on the computational side, like the encapsulation of variables in Fortran types (*i.e.* structures or packs of variables) or to make standard the use of 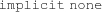, a good programming practice, which was not followed in *SHADOW2*. The new changes will be implemented gradually, maintaining a balance between user support and new development.

## 4. The new *SHADOW3* code structure

### 4.1. Modules and variables

The new *SHADOW* source uses the modular approach made available by Fortran 90. This was a major Fortran upgrade from Fortran 77. Since then, Fortran 95 was a minor release, and Fortran 2003 (and its Fortran 2008 update) introduced new concepts in object-oriented programming not yet exploited in *SHADOW*. The *SHADOW* functions and subroutines have been distributed in a few modules. The *SHADOW* kernel consists of:

(i) 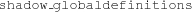: basic definitions used everywhere, like variable kind, *etc*.

(ii) 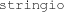: some string manipulation tools.

(iii) 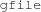: a new Fortran module to manipulate files with list of input/output variables (called *g-files* in *SHADOW*).

(iv) 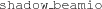: routines to access to binary beam files (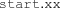, 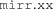, 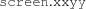).

(v) 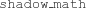: mathematical tools.

(vi) 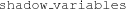: definition of variables and Fortran types used by the kernel, plus the routines to manipulate them.

(vii) 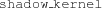: contains the global variables (ex-common blocks) and the routines in the *SHADOW* kernel.

In addition to the kernel, *SHADOW3* contains the following:

(i) 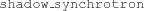: synchrotron sources (bending magnets, wiggler and undulators).

(ii) 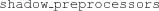: pre-processors, like 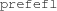 or 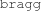.

(iii) 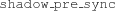: pre-processors for 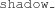 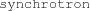.

(iv) 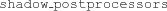: post-processors, like 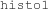 or 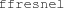.

(v) Main programs: all *SHADOW3* is included into a single command line executable: 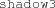. All pre- and post-processors are included in this executable. In addition, 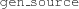 and 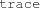 are also created for being 100% compatible with the old versions.

**Figure 1:**
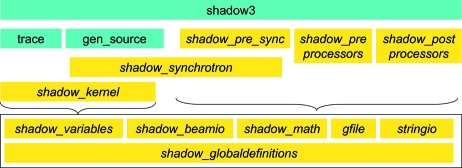
Module dependencies in *SHADOW3*. Modules are in light grey boxes (yellow online) and main programs in dark grey boxes (green online).

The modules map is schematized in Fig. 1 ▶. In the old *SHADOW*, the variables were imported from the 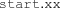 files. For that, *SHADOW1* used 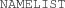, a non-standard Fortran 77 extension, whereas *SHADOW2* used a set of C routines linked to Fortran. Once imported, the variables were sent directly into common blocks. In *SHADOW3*, the variables defined in the 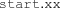 files are now put into three levels: (i) from the files, they are read into a 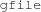 Fortran type which is just a table of variable names and values, (ii) these values are then copied to two Fortran types, called 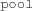 (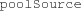 and 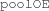), which are binded to C, and (iii) the pool variables are copied to global variables in 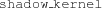. This three-level scheme may appear complex, but it was considered necessary to gain in modularity and portability, and to be able to define an API. In the future, the copy of the pool variables into global variables in 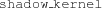 will be removed, thus directly using the pool variables in the computation.

In the old version, *SHADOW* sources (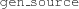) dealt with all kinds of sources (geometrical, bending magnet, wiggler and undulator). The new version contain three separated identities in the code:

(i) 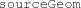: a routine in the 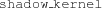 module used to generate geometrical sources.

(ii) 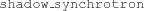: an independent Fortran module for bending-magnet and insertion devices sources. It has been separated from the kernel. The synchrotron pre-processors are in a new module 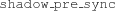. The old 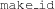 script is now fully implemented in the 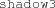 executable.

(iii) 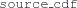: a new generic source. It reads the characteristics from external files, *e.g.* produced by external programs [*SRW* (Chubar & Elleaume, 1998 ▶), *SPECTRA* (Tanaka & Kitamura, 2001 ▶), *XOP* (Sanchez del Rio & Dejus, 2004 ▶), *etc.*] and sample rays using these distributions. This is still under development and will be available in *SHADOW3.1*.

*SHADOW* has an optical library populated with information on cross sections and scattering factors which is used to obtain the material properties, refraction index and crystal structure factors. The *SHADOW* kernel does not directly call the optical library, but only reads material data files filled with physical constants that were created using pre-processors linked to the optical library. Thus, the optical library is external to the kernel, simplifying the scheme. The old optical library is still used, but new libraries with updated values are also provided. In addition, the pre-processors can also be built using other well established optical libraries, such as 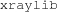 (Brunetti *et al.*, 2004 ▶). The two principal graphic post-processors, 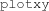 and 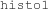, have been modified to produce gnuplot graphics (see http://www.gnuplot.info/), a powerful free and multiplatform graphics package.

### 4.2. The *SHADOW3* API

Ray-tracing calculations can sometimes be tedious owing to the high number of beamline parameters and combinations to analyze. The optimization of a single parameter can be done manually or automatically by sampling the parameter and performing a loop of *SHADOW* runs. However, to simultaneously optimize several parameters, it is obvious that an exhaustive search of the best value is impractical so the only solution, at present, is the heuristic intuition of the developer. An automation of such a global search of optimal parameters can be done using genetic algorithms, simulating annealing or any other global optimization technique. An API is needed for such techniques, and for allowing the programmer to create loops, macros and scripts with *SHADOW*.

**Figure 2:**
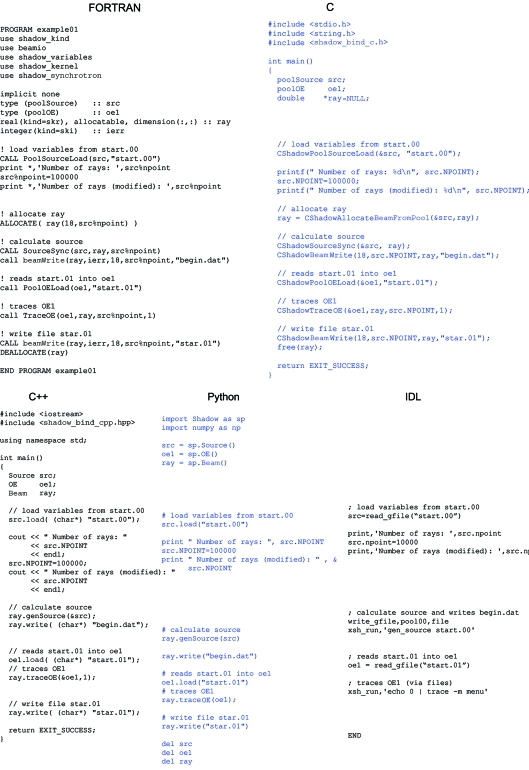
Example of a simple *SHADOW3* main program in Fortran, C, C++, Python and IDL.

*SHADOW3* has been first interfaced to C, with not only the idea of writing new programs in C, but also as an intermediate step to using other higher-level languages, such as Python (http://www.python.org/) and IDL (http://www.ittvis.com/). The memory allocation of the beam (a collection of rays) is an important issue of the API. This must be done at the main level (Fortran, C, *etc.*). The API module imports a reduced number of procedures from Fortran, all of them considered functions in C (the *exposed* functions). Although it is technically possible to directly use the Fortran functions from C, it creates problems for a C programmer, because in Fortran all the arguments are passed by reference and the definition of strings is not compatible. Therefore, a solution has been chosen where only a set of C-functions (named with the prefix 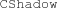) and C-structures access the Fortran procedures and types. These C-functions are documented, and their names mimic the Fortran counterparts. This C-API also puts the basis for a C++ layer that utilizes the C-structures and the exposed functions to define classes. *Source*, *Optical-element* and *Beam* are classes in the C++ layer. The C-API is also the ground level for developing Python classes. Python comes with an advantage: it is a script language, thus the user has direct access to the *SHADOW* kernel permitting any batch or macro programming without any compilation. *SHADOW3* has also been interfaced to IDL, because the *ShadowVUI* GUI is written in IDL. Fig. 2 ▶ shows an example of a simple main code to run a source and a single optical element written in Fortran, C, C++, Python and IDL.

### 4.3. GUIs

**Figure 3:**
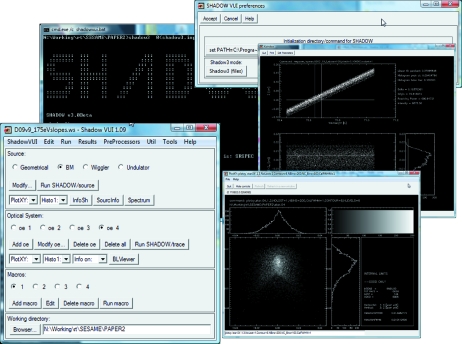
Window of *ShadowVUI* running *SHADOW3*.

*SHADOW3* is delivered without a GUI. The old TCL/TK *SHADOW* GUI shipped with previous versions of *SHADOW* is now obsolete and has been discontinued. Work is invested in improving and updating *ShadowVUI*, an IDL-based GUI that is available free to users as part of the *XOP* package. *ShadowVUI* has been adapted to *SHADOW3* and can be configured to either select *SHADOW3* or the previous *SHADOW2*. The *ShadowVUI* also has powerful scripting capabilities, using script commands based on IDL and calling directly the IDL functions used in *ShadowVUI*. See Fig. 3 ▶ for an example. The IDL scripting will slowly migrate to the more powerful Python scripting.

### 4.4. *SHADOW3* distribution

Two servers have been set for *SHADOW3*. The first one, http://ftp.esrf.eu/pub/scisoft/shadow3/, is used for downloading the *SHADOW3* binaries compiled for different platforms (Windows, MacOS and Linux) and related documentation. It is intended for use by users only interested in running *SHADOW*. The *SHADOW* source code is distributed and managed using the version control system *git* (http://www.git-scm.com/). Sources can be downloaded by any user using the command: ‘git clone git://git.epn-campus.eu/repositories/shadow3/’. The http://forge.epn-campus.eu/projects/shadow3/ server contains the complete history and full revision tracking of *SHADOW3* plus other services like an issue or bug tracker and a developer forum (wiki). Any user can see it but, for entering new issues and uploading new code, the developers must apply for an account. We propose that any registered developer could upload new applications, macros, post-processors, *etc.*, but modifications in the *SHADOW* kernel will be subject to the acceptance of the manager. However, *git* is flexible enough to allow any registered developer to create a new code *branch* in the server, that can be incorporated in the *master branch* by the manager.

## 5. Examples

The main objective of *SHADOW3* is to provide the full functionality of previous versions, so there is no new development from the point of view of models and algorithms. The installation is made much more simplified by the use of a single executable 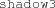 that allows calling the main commands (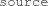 and 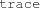) plus the pre- and post-processors in a simple and integrated environment.

The *SHADOW* primer, available to users since 1994, explained in detail all aspects of the code that the user needed for setting the calculations. It contains the basic explanations of the *SHADOW* reference system, and explains in great detail how to run in command mode using several important examples, like the creation of geometrical and synchrotron (bending-magnet) sources, mirror focusing, and grating and crystal monochromators. We have updated the *SHADOW* primer to *SHADOW3*, including the new features, mainly the graphics output now based in 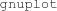. Moreover, all the examples are also provided as text input files, thus it is very easy to rerun the examples using these files as standard input for *SHADOW3*. In addition, a script is available to run all the examples in the primer from a single command. The new primer and input files are available from the *SHADOW3* repository mentioned before. They also constitute a collection of tests to check back-compatibility of future upgrades.

Good practice when performing Monte Carlo simulations is to complement the calculation of a given parameter *P* with error estimation. Errors can be estimated in two ways. The first uses the fact that the Monte Carlo estimate is asymptotically normally distributed (approaches a Gaussian density) (James, 1980 ▶). Therefore, if one performs *M* *SHADOW* runs with *N* rays per run, one obtains *M* independent results for *P* (*p*_*i*_, *i* = 1,…, *M*) that can be used for computing the average 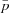 and its standard deviation, a good estimator of the error. It is also possible to calculate the value and the error of *P* from a single *SHADOW* run of *N* rays. Following James (1980 ▶), in order to calculate the average and the standard distribution of a scored parameter *P* it is necessary to accumulate: the sum of the weights *w* of the scored rays, *p* = 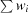, where the sum extends to all scored rays; the sum of the squares of the weights, *q* = 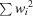; and the total number of rays, *N*. The final total counting for the parameter *P* (plus and minus one standard deviation) is
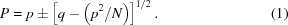
Consistently, in the case that one scores non-weighted rays (*i.e.* ‘reflectivity’ is not set in *SHADOW*, in other words, we are ‘counting rays’), every ray has the same weight equal to 1, so *q* = *p* and, for 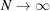, one obtains *P* = 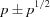, an expression typically used with counter detectors (Poisson statistics). The error estimation based on this idea has been implemented in post-processors like 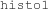 and 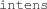.

**Figure 4:**
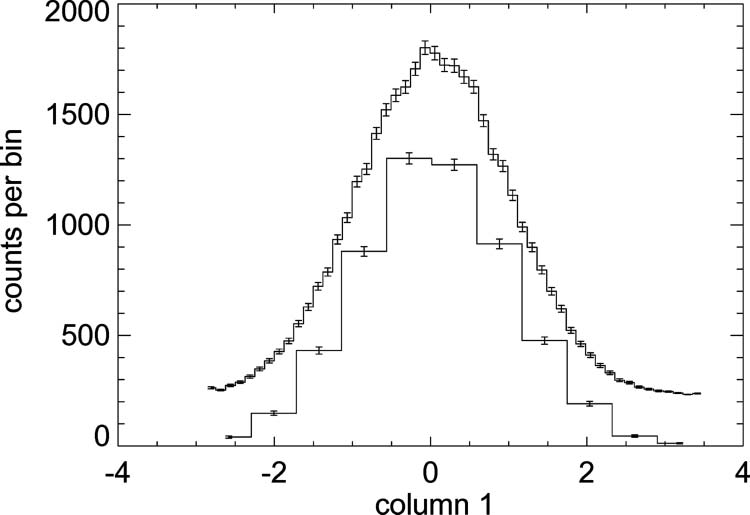
Example of a *SHADOW* loop to accumulate counts into a histogram (see code in Appendix *A* [App appa]). The two graphs are the results for the system defined in the *SHADOW* primer (ch. 6.3) with different histogram resolution, 11 and 51 bins, that needed 10000 and 53000 rays, respectively, for reducing the standard deviation to less than 2%. The histogram with 51 bins is shifted for clarity.

Both ways of computing errors are easily applied to *SHADOW3*, that can now trace any number of rays in a single run, limited only by the computer memory. In many cases it is prefereable to perform *M* runs with *N* rays and accumulate the results, rather than performing a single run with *M × N* rays. This is more efficient from the computer’s point of view, as not all the rays are stored in the memory at the same time (and perhaps in a large file) but only a small number of rays are traced at once, then the required results are scored, before returning and processing the next run that will calculate another bunch of rays and accumulate the results. Fig. 4 ▶ shows an example where *SHADOW* runs in a loop and accumulates results in a histogram. The loop continues until a ‘quality’ criterium is reached. In this case, that error (standard deviation) in intensity at the centre of the histogram must be less than 2%. This program runs very fast, as no files are written. Obviously, more runs are needed if the number of bins of the histogram is increased.

**Figure 5:**
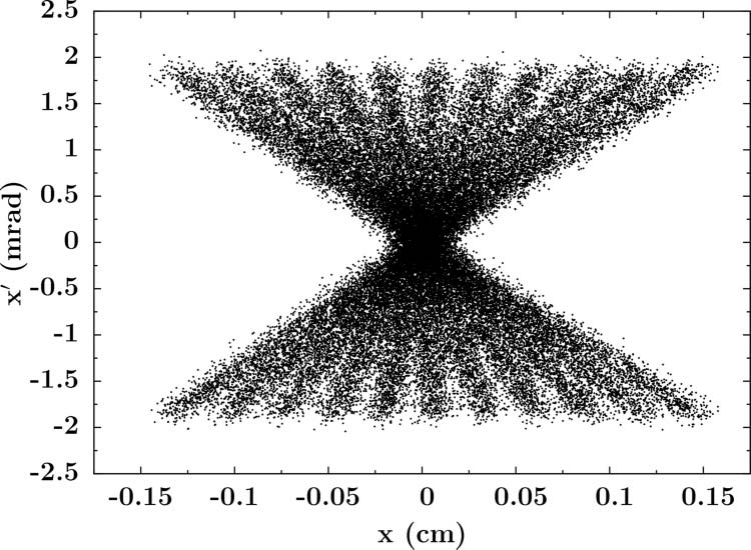
Plot of the horizontal phase space for a wiggler (ID17 at the ESRF) with 11 periods of 0.15 m length, *K* = 22.3 and electron beam energy of 6.04 GeV.

The power of the *SHADOW3* API also opens the door to combine *SHADOW3* with other large simulation packages. For instance, the combination of *SHADOW3* with the Bmad library (http://www.lepp.cornell.edu/~dcs/bmad) for relativistic charged-particle dynamics simulations in storage rings is being considered. The *SHADOW3* code for accurate simulation of the wiggler source has been used together with the *PENELOPE* code (Salvat *et al.*, 2008 ▶). This code performs Monte Carlo transport of electrons and photons in matter, which is of great interest to medical physics for dosimetry calculations. Experiment planning of synchrotron microbeam radiation therapy requires an accurate description of the X-ray source phase space. *SHADOW3* is used for creating an exact description of the wiggler source, which has a complex geometry (see Fig. 5 ▶) not easily implemented by geometric models. The simulated photons (*i.e.* the rays created by *SHADOW3*) are then input to *PENELOPE* for calculating the deposited energy dose in a phantom or tissue.

**Figure 6:**
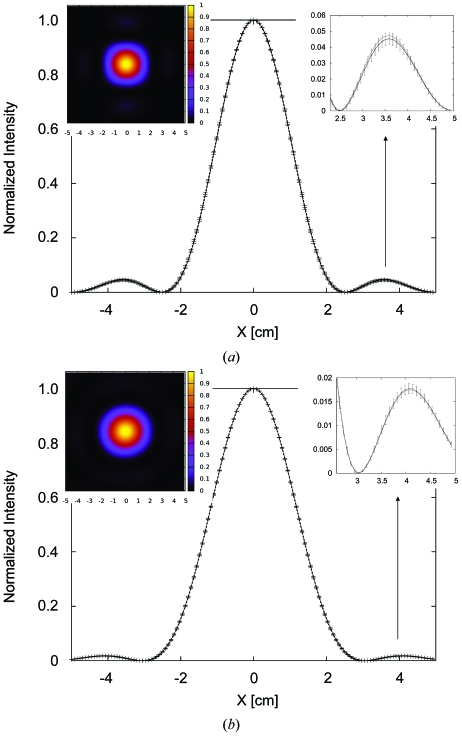
Fraunhoffer diffraction pattern of a square aperture (*a*) and circular aperture (*b*) as calculated by the 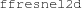 post-processor. A coherent Monte Carlo source of 1024 rays of 1 nm wavelength was sent to illuminate the 4 µm-wide slit. The detector is placed 100 m downstream from the slit. The image on the detector is sampled by 128 × 128 pixels. The *SHADOW* results of averaging 64 runs (dotted line), from which standard deviations are calculated, have been compared with the analytic theoretical pattern (solid line) with excellent agreement.

*SHADOW* can perform wave optics propagation of the beam from an image plane to a detector plane, using the 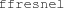 post-processor. It requires the propagation of every ray to each point in the detector plane by applying the Fresnel–Kirchhoff integral. In old versions, this was usually done for computing one-dimensional intensity profiles. With the new version, it is possible to create full two-dimensional interference/diffraction patterns with the new 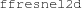 routine. An example is shown in Fig. 6 ▶.

## 6. The future of *SHADOW*

The *SHADOW* code has always been tied to the bright and strong personality of its main author, Professor Franco Cerrina, who passed away on 12 July 2010. The work presented here is just a first step in an ambitious plan of continued maintenance and development that has accompanied the evolution of the synchrotron radiation facilities in the last 25 years. *SHADOW3* is a first step in cleaning the kernel and preparing a programming platform that will face exciting new developments, such as: (i) propagation of coherent and partially coherent X-ray beams, *e.g.* for interference and phase contrast applications; (ii) simulation of samples for calculating instrumental functions, and to be used as sources for ray-tracing analysers; (iii) evolving from a macroscopic model for dealing with the optical elements, to a microscopic model, where a full Monte Carlo analysis is performed within the optical elements; (iv) intensive calculations with a high number of rays using variance reduction techniques and global optimization methods. There is also the urgent need for some applications; for instance, the full implementation of compound refractive lenses, ability to treat any crystal structure, introduction of roughness in multilayers, implementation of laterally graded multilayers, beam penetration simulation inside diffractive elements (multilayers and crystals), computation of the grating efficiency, *etc*. The development of *SHADOW* to match these challenging developments is uncertain, as now we lack the guidance and direction of Franco Cerrina. We firmly believe that the survival of *SHADOW* and its future development cannot proceed without the implication and engagement of the synchrotron laboratories. *SHADOW* is a unique software created and targeted to synchrotron optics, and it should be the responsibility (and the interest) of the synchrotron laboratories to guarantee a future that will certainly benefit them. In the future we will discuss and organize plans for its continuation and development, merging efforts from many synchrotron laboratories.

## Figures and Tables

**Figure 7:**
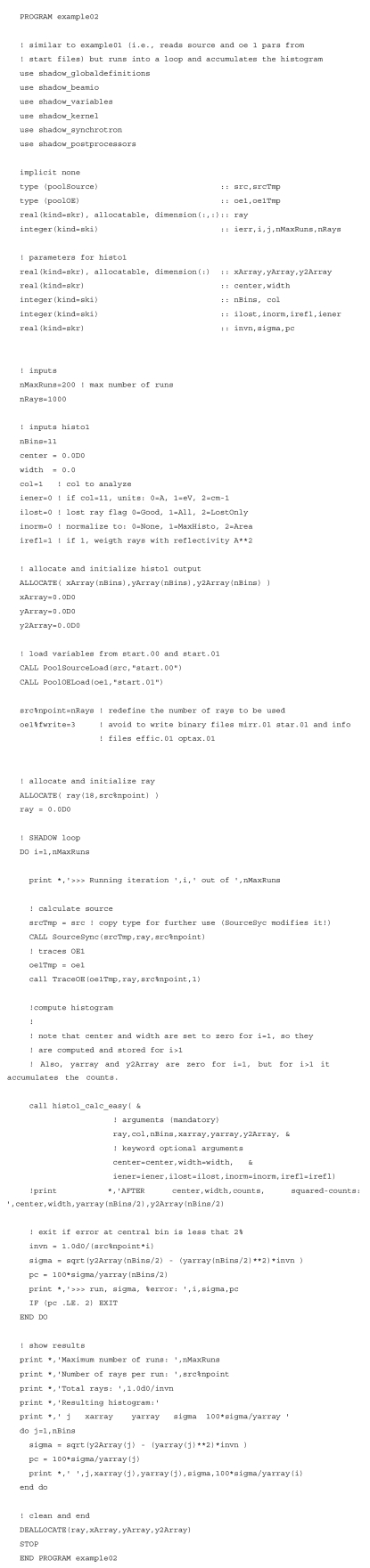
The code used for Fig. 4 ▶.

**Figure 8:**
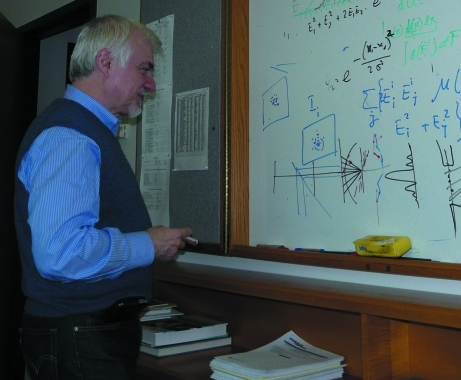
Franco Cerrina looking at new algorithms to be implemented in *SHADOW*. Photograph taken by M. Sanchez del Rio on 17 March 2009.

## Appendix A

The code used for Fig. 4 ▶ is shown below in Fig. 7 ▶.
